# Advances in Cross-Coupling Reactions

**DOI:** 10.3390/molecules25194500

**Published:** 2020-10-01

**Authors:** José Pérez Sestelo, Luis A. Sarandeses

**Affiliations:** Centro de Investigaciones Científicas Avanzadas (CICA) and Departamento de Química, Universidade da Coruña, E-15071 A Coruña, Spain

Cross-coupling reactions stand among the most important reactions in chemistry [1,2]. Nowadays, they are a highly valuable synthetic tool used for the preparation of a wide variety of organic compounds, from natural and synthetic bioactive compounds to new organic materials, in all fields of chemistry [3]. Almost 50 years from its discovery, the research in this topic remains active, and important progresses are accomplished every year. For this reason, we believe that a Special Issue on this topic is of general interest for the chemistry community.

Advances in cross-coupling reactions have been developed with the aim to expand the synthetic utility of the methodology, through the involvement of new components, reaction conditions, and therefore, novel synthetic applications [4]. Although initially the term “cross-coupling” referred to the reaction of an organometallic reagent with an unsaturated organic halide or pseudohalide under transition metal catalysis, currently the definition is much more general and applies to reactions involving other components, conditions, and more complex synthetic transformations. In addition to the well-known and recognized cross-coupling reactions using organoboron (Suzuki-Miyaura), organotin (Stille), organozinc (Negishi), or organosilicon (Hiyama) nucleophiles, reactions involving other organometallic reagents such as organoindium [5,6], organolithium [7], and Grignard reagents [8] are now useful synthetic alternatives. Moreover, a wide range of carbon nucleophiles, from stabilized carbanions such as enolates and derivatives to neutral species, are also efficiently used [9]. Interestingly, cross-coupling reactions involving transition metal-catalyzed C–H activation have also been described [10]. They also can be used to form carbon–heteroatom bonds with heteronucleophiles such as amines and alcohols (Buchwald-Hartwig), among others [11,12].

On the other hand, the set of coupling partners used as electrophiles have been widely enlarged, from the classical organic halides and sulfonates to substrates with higher C–O bond dissociation energy, such as ethers and carbamates [13] and to those involving the cleavage of C–N bonds, such as amine and nitro derivatives [14,15]. More recently, carboxylic acid derivatives have been also incorporated since decarboxylative, and related processes reveal as a powerful method to generate electrophilic species with application in modern coupling reactions [16,17].

The discovery of novel metal catalysts and ligands is also a topic of continuous interest. From the original palladium complexes, the use of other Earth-abundant first-row transition-metals as catalysts such as iron, cobalt, or copper has emerged as alternatives in coupling reactions [18]. In this sense, nickel catalysts have been shown especially useful due to its high nucleophilicity and number of oxidation states [19]. In relation with the use of nickel, photoredox catalysis [20,21] and, in general, coupling processes involving radical species have gained of particular relevance and constitute an area in continuous expansion [22]. Additionally, the modulation of the catalytic activity through the design of ligands with singular steric or electronic properties provides an increasing number of possibilities. Of particular relevance are biaryl phosphines [23], and the incorporation of carbenes as ligands [24]. This research has allowed us to improve the efficiency of the coupling reactions with lower catalyst loading, lower reaction temperatures, and shorter reaction times.

Another important challenge in cross-coupling reactions is to perform alkyl–alkyl couplings. These transformations have been traditionally hampered due to the undesired β-H elimination side reaction. However, recent advances in nickel catalysis have allowed the development of remarkable examples of such transformations. Even more importantly, some of the newly developed catalytic systems have been applied to enantioselective reactions [25].

All these advances have facilitated the implementation of cross-coupling reactions in industry. In this sense, a complete set of reaction conditions have been developed from aqueous or anhydrous to homogeneous or heterogeneous systems in small or large scale. In addition, novel technologies, such as solid-phase coupling reactions and flow-chemistry technology are being used in the synthesis of bulk chemicals and pharmaceutical products.

In this Special Issue, some representative examples of recent advances in cross-coupling reactions have been collected in the form of reviews, articles, and communications. These contributions cover different topics, from new methodologies and reaction conditions, some synthetic alternatives, new metal ligands, and synthetic applications for new pharmaceutical compounds and organic materials.

In a short review, Sotomayor, Lete et al. present the recent advances in the synthesis of diarylketones through a Pd(II)-catalyzed acylation of (hetero)arenes and the coupling reaction with aldehydes under oxidative conditions [26]. This synthetic transformation represents an alternative to the traditional coupling using aryl organometallics and acyl halides. As an example of metal-free coupling reactions, Trofimov et al. present the inverse Sonogashira coupling between pyrroles and haloalkynes for the synthesis of 2-alkynylpyrrols, a unit present in many bioactive molecules [27]. This synthetic approach overcomes some limitations of the Sonogashira coupling when electron-rich heterocycles are employed. In a related procedure, and as alternative to the traditional cross-coupling protocols, Zhang et al. present the coupling of indoles with diverse C–H nucleophiles under oxidative dearomative cross-dehydrogenative conditions. As a result, 2,2-disubstituted indolin-3-ones are obtained [28].

The high chemoselectivity exhibited by transition-metal-catalyzed cross-coupling reactions can be exploited to develop various synthetic transformations using one-pot procedures. In this issue, Malezcka, Smith et al. describe the efficient combination of the regioselective iridium-catalyzed C–H borylation of aryl halides with the Sonogashira coupling [29]. Interestingly, the coupling reaction takes place selectively at the carbon–halogen bond allowing the preparation of novel alkynyl boron reagents. In a related article, Chotana et al. report a sequential iridium-catalyzed borylation of NH-free pyrroles followed by a Suzuki-Miyaura reaction [30].

As previously stated, cross-coupling reactions are valuable synthetic tools for the synthesis of pharmacologically active compounds. With a personal view form the pharmaceutical industry, Blanco and Buskes review the most relevant contributions of Suzuki-Miyaura and Buchwald-Hartwig coupling reactions to the synthesis of bioactive compounds [31]. As a communication, Beller et al. report the synthesis of aryl propionic acids, a common structural motif in medicinal chemistry, through combination of a palladium-catalyzed Heck coupling reaction with a rhodium-catalyzed hydroformylation [32].

The synthetic utility of cross-coupling reactions for the synthesis of medium size rings is covered by Gulea et al. [33]. In this review, cross-coupling reactions are shown as a valuable synthetic tool to overcome classical methods and as alternatives to other metal-catalyzed reactions such as alkene metathesis.

The Stille and Suzuki coupling reactions are used by Nikitin et al. for the synthesis of new molecular machines based on sterically hindered anthracenyl trypticenyl units [34]. Zani et al. show the utility of cross-coupling reactions for the synthesis of new organic dyes containing the indigo core, being the derivatization efficiently accomplished by a Stille coupling in the last step of the synthesis [35].

The catalytic activity of palladium(II)-salan complexes in Suzuki-Miyaura cross-coupling reactions is studied by Udvardy, Joó et al. as an alternative to classical phosphines, showing that salan ligands can be used in water and air to perform cross-coupling reactions [36].

In summary, cross-coupling reactions constitute one of the most relevant methods in modern organic chemistry and have allowed many new transformations in this science. The impact of these reactions in academia and industry is profound, and this continuous research tends to develop more sustainable, economic, and efficient processes. Contributions from this Special Issue in *Molecules* try to meet this end.

Finally, we want to thank the authors for their contributions to this Special Issue, all the reviewers for their work evaluating the submitted articles, and the editorial staff of *Molecules*, especially the Assistant Editor of the journal, Emity Wang, for her kind assistance during the preparation of this Special Issue.
